# Effects of lower limb length discrepancy on spinopelvic compensation following total hip arthroplasty in patients with developmental dysplasia of the hip

**DOI:** 10.1186/s13018-024-04816-7

**Published:** 2024-06-08

**Authors:** Tong Li, Yifei Li, Jiaxiang Gao, Ruichen Ma, Qidong Zhang, Weiguo Wang

**Affiliations:** 1 Department of Orthopedics, China-Japan Friendship Hospital, Beijing, China; 2 15 th Department, Plastic Surgery Hospital, Chinese Academy of Medical Sciences and Peking Union Medical College, Beijing, China

**Keywords:** Lower limb length discrepancy (LLLD), Developmental dysplasia of the hip (DDH), Total hip arthroplasty (THA), Coronal decompensation (CD), Compensation

## Abstract

**Background:**

Limited research has examined the impact of lower limb length discrepancy (LLLD) alteration on spinopelvic compensation in individuals with developmental dysplasia of the hip (DDH). This study aimed to investigate the effects of LLLD on spinopelvic compensation following total hip arthroplasty (THA) and elucidate the complex biomechanical adaptations in the spinopelvic structures.

**Methods:**

A retrospective review of DDH patients undergoing THA from January 2014 to December 2021 categorized individuals with Crowe type I and II into the low dislocation group (LDG, *n* = 94) and those with Crowe type III and IV into the high dislocation group (HDG, *n* = 43). Demographic data, as well as preoperative, postoperative, and last follow-up imaging data, including lower limb length (LLL), sacral obliquity (SO), iliac obliquity (IO), hip obliquity (HO), Cobb angle, apical vertebral translation (AVT), and coronal decompensation (CD), were collected for analysis.

**Results:**

Patients in the LDG had a significantly higher surgical age and shorter disease duration (*P*<0.05). In LDG, patients exhibited substantial postoperative reductions in LLLD, SO, IO, and HO (*P*<0.05), while Cobb Angle, AVT, and CD showed no statistically significant changes (*P*>0.05). The variation in LLLD correlated significantly with the variations in SO, IO, and HO (*P*<0.05). Postoperative outcomes in the HDG demonstrated marked decreases in LLLD, SO, IO, HO, and CD (*P*<0.05), with no significant change in Cobb angle and AVT (*P*>0.05). The variation in LLLD correlated significantly with the variations in SO, IO, HO, and CD (*P*<0.05).

**Conclusions:**

THA effectively reduces LLLD in patients with DDH, and the variation in LLLD correlates meaningfully with the recovery of spinopelvic compensatory mechanisms.

## Background

Developmental dysplasia of the hip (DDH) encompasses a spectrum of developmental disorders affecting the hip from early childhood [1]. While lameness in childhood can be corrected through interventions such as cast immobilization and osteotomies, many individuals with DDH face neglect [2]. Residual DDH that occurs in adulthood is often accompanied by pain and early onset of osteoarthritis [3], which may ultimately require total hip arthroplasty (THA) to achieve proper alignment of the femoral head within the acetabulum [4].

Lower limb length discrepancy (LLLD) is a critical factor that significantly influences surgical outcomes, especially for those with severe dislocation [5, 6]. Therefore, it necessitates meticulous attention during preoperative planning and subsequent stages of postoperative functional recovery [7, 8]. Patients with DDH frequently encounter abnormal functional lower limb length (LLL) due to the abnormal position of the femoral head, morphological changes in the proximal femur [9], and valgus deformity of the knee joint [10], which can lead to secondary pelvic obliquity and scoliosis [11]. While the effectiveness of THA in improving LLL and its impact on pelvic obliquity have been recognized [12], there is a dearth of research on the impact of THA on scoliosis. The complex interaction between LLLD and compensatory mechanisms of the pelvis and spine in DDH patients remains an ongoing subject of investigation.

The presence of scoliosis, often associated with severe DDH, can result in coronal imbalance of the spine and exacerbate strain on the lower limb joints during walking [13, 14]. This underscores the importance of scoliosis recovery in prolonging the lifespan of joint prostheses. Therefore, it is crucial to comprehend the intricate connections between LLLD, pelvic obliquity, scoliosis, and their postoperative changes to improve surgical strategies and optimize long-term outcomes for this patient population.

Limited research has examined the impact of LLLD alterations on spinopelvic compensation in individuals with DDH. Through a retrospective analysis of preoperative and postoperative imaging data, this study aimed to comprehensively explore the effects of LLLD on spinopelvic compensation following THA and elucidate the complex biomechanical adaptations in the spinopelvic structures.

## Methods

### Study designs

Prior to the study, approval was obtained from the ethical committee of our hospital, and all participants provided written informed consent before enrollment. Between January 2014 and December 2021, a total of 431 patients underwent THA conducted by a senior surgeon at our institution, with 223 of them diagnosed with DDH. The inclusion criteria were as follows: (1) confirmation of unilateral DDH through preoperative hip X-ray (center-edge (CE) angle<20° or Sharp angle>45°), (2) completion of a unilateral, primary, and cementless THA on the affected side; (3) absence of hip disease on the contralateral side, (4) no history of hip or spinal surgery prior to THA, and (5) follow-up period of more than two years. Based on these criteria, 148 out of 223 patients were included. The exclusion criteria included: (1) history of other major arthropathy (e.g., rheumatoid arthritis or ankylosing spondylitis), childhood hip disease (e.g., Legg-Calvé-Perthes or slipped femoral epiphysis), or neurological disorder (e.g., polio) (*n* = 4); (2) previous surgeries on the spine, pelvis, hip joint, or other parts that might affect the LLLD or spinopelvic parameters (*n* = 4); and (3) DDH combined with osteonecrosis, fracture, or infection (*n* = 3). Consequently, 137 out of 148 patients were retrospectively included in this study. Patients were further stratified based on the preoperative Crowe classification into the low dislocation group (LDG, *n* = 94) for Crowe type I and II, and the high dislocation group (HDG, *n* = 43) for Crowe type III and IV. This robust methodology ensures a focused investigation into the impact of THA on LLLD and spinopelvic parameters in a well-defined cohort of patients with unilateral DDH.

### Data collection

**Fig. 1:**
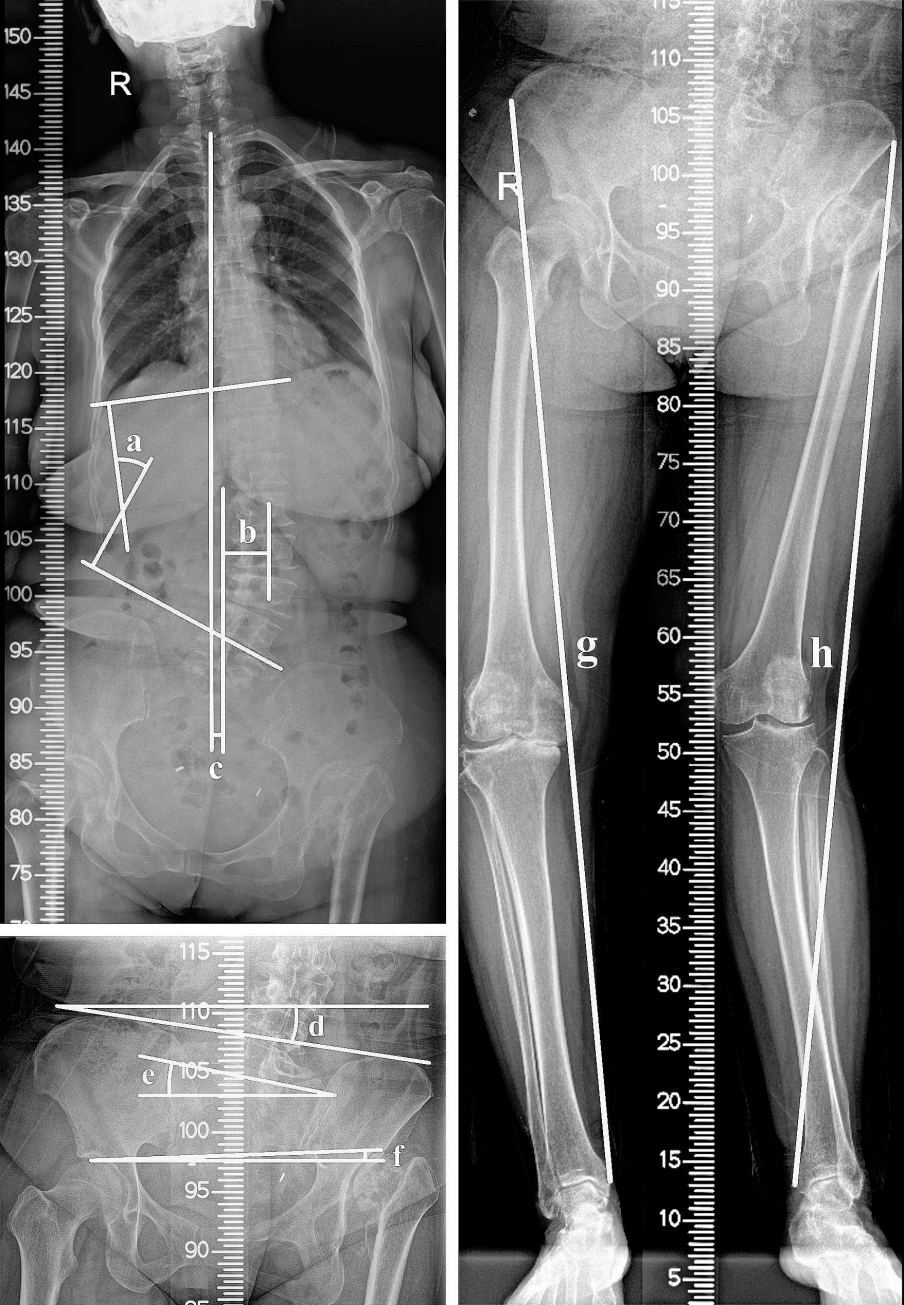
Measurements of radiographic evaluations. **(a)** Cobb angle; **(b)** Apical vertebral translation (AVT); **(c)** Coronal decompensation (CD); **(d)** Sacral obliquity (SO); **(e)** Iliac obliquity (IO); **(f)** Hip obliquity (HO); **(g)** Healthy lower limb length (HLLL); **(h)** Affected lower limb length (ALLL)

Patient data, including age, gender, BMI, duration of disease, and postoperative complications were collected to analyze their clinical characteristics. Preoperative, postoperative, and last follow-up radiographic evaluations were conducted for all participants using full-length anteroposterior radiographs of both lower limbs and the spine (Fig. 1). The full-length anteroposterior radiographs of both lower limbs were utilized to assess LLL, sacral obliquity (SO), iliac obliquity (IO), and hip obliquity (HO). LLL was measured as the distance from the anterior superior iliac spine to the tip of the medial ankle. Affected lower limb length (ALLL), healthy lower limb length (HLLL), and LLLD offered valuable insights into limb asymmetry. SO, IO, and HO were assessed, with SO determined by the angle between a straight line connecting two transition points between the sacral wing and the S1 superior articular process and the horizontal line, IO calculated as the angle between the line connecting the iliac crests and the horizontal line, and HO measured as the angle between the highest point of the femoral head and the horizontal line. A positive value indicated an obliquity toward the healthy side, while a negative value indicated an obliquity toward the diseased side. The full-length anteroposterior radiographs of the spine were utilized to evaluate Cobb angle, apical vertebral translation (AVT), and coronal decompensation (CD). Cobb angle gauged the extent of curvature by determining the angle between the upper endplate of the vertebra above the curve and the lower endplate of the vertebra below the curve. AVT measured the horizontal shift of the apical vertebra from the central sacral vertical line, indicating lateral displacement. CD assessed the C7 plumb line from the central sacral vertical line, providing insights into postural imbalance in the coronal plane. The digital images were stored and retrieved for measurement using the Carestream Vue HIMS system. To minimize potential systematic bias, the measurements were independently conducted by two researchers and then averaged.

### Surgical technique

The patient was positioned laterally for the posterior approach, with the incision originating below the greater trochanter and curving upward towards the posterior pelvis. Incisions were made in the short external rotators and piriformis muscles at their attachment points on the greater trochanter, which were clearly marked for subsequent repair. Employing a pendulum saw after incising the pseudojoint capsule facilitated bone dissection, and the femoral head was extracted using a head extractor. The pseudojoint capsule, surrounding tissues, gluteus maximus insertion, and iliotibial band were completely released. Gently pulling the femur forward and releasing tissue downward allowed access to the true acetabulum and proximal femur. Hohmann retractors were strategically placed around the acetabulum to provide optimal exposure, and a posterior retractor was used to retract the posterior joint capsule for visualization of the acetabulum. An acetabulum file was used to refine size of the acetabulum, guided by soft tissue landmarks to verify anteversion and inclination, ensuring accurate placement of the cementless Pinnacle acetabular cup (DePuy Synthes) with a ceramic liner. The appropriate cup size was chosen, with a focus on achieving at least 70% coverage to maintain cup stability. For patients with low dislocation and a generally normal femoral medullary cavity morphology, the femoral bone marrow cavity was appropriately extended, and a fitting cementless Corail femoral stem (DePuy Synthes) was chosen and implanted. In cases of high dislocation or abnormal medullary cavity morphology, a fitting cementless S-ROM stem (Depuy Synthes) was selected to restore the proper anteversion of the femur. When the femoral head was planned to place back into the true acetabulum, the ALLL was significantly extended, posing a risk of being unable to reduce the hip or of neurovascular injury after reduction. If deemed necessary, an appropriate subtrochanteric osteotomy was conducted to facilitate the proper restoration of the femoral head into its true acetabulum [15]. Finally, repair of the short external rotators and the posterior capsule was accomplished through transosseous bone tunnels in the proximal femur.

### Statistical analysis

The data was analyzed using SPSS version 26.0. A comparative assessment of each parameter was undertaken preoperatively, postoperatively, and at the last follow-up, with the variation from preoperative to the last follow-up denoted as Δ. Interobserver reliability was qualified using a single-measure (2-way random) intraclass correlation coefficient, with values greater than 0.75 indicating satisfactory reliability. Measurement data that followed a normal distribution were presented as mean±standard deviation (s), and *t*-tests and variance analysis were used for inter-group and intra-group comparisons. Frequency analysis was used for statistical descriptions of counting data, and comparisons were made using the *Chi*-square test. The *Pearson* correlation coefficient was employed to examine the relationships between the variation of LLLD and the variations of coronal spinopelvic parameters. A significance level of α<0.05 was established to determine statistical significance.

## Results

Regarding baseline characteristics, patients in the LDG exhibited a significantly higher surgical age compared to those in the HDG (58.6±8.2 years in LDG vs. 40.0±11.7 years in HDG, *P*<0.001, Table 1). However, no statistical differences were observed between the two groups in terms of gender, BMI, disease duration, or length of follow-up period (*P*>0.05, Table 1). Intraclass correlations of radiographic measurements were shown in Table 2.

**Table 1:** Basic characteristics of patients in the low dislocation group compared with the high dislocation group

Variable	LDG (*n* = 94)	HDG (*n* = 43)	*P*-value	95% CI for difference in group means
Gender (Men/Women)	38/56	14/29	0.379	
Age (year)	58.6 ± 8.2	40.0 ± 11.7	< 0.001**	14.73 to 22.64
BMI (kg/m^2^)	25.0 ± 1.8	25.5 ± 2.1	0.182	-1.15 to 0.22
Disease duration (month)	41.5 ± 14.1	47.0 ±13.6	0.035*	-10.55 to -0.40
Follow-up period (month)	40.0 ± 10.6	38.1 ± 10.3	0.324	-1.92 to 5.75

LDG: low dislocation group; HDG: high dislocation group; BMI: body mass index. *: Significant at *P*<0.05, **: Significant at *P*<0.001.

**Table 2:** Intraclass correlation coefficient for radiographic measurements

Variable		Intraclass correlation	95% CI	*P*-value
HLLL (mm)	Pre-operative	0.994	0.991 to 0.995	< 0.001**
	Post-operative	0.992	0.989 to 0.994	< 0.001**
	Last follow-up	0.990	0.985 to 0.993	< 0.001**
ALLL (mm)	Pre-operative	0.992	0.989 to 0.994	< 0.001**
	Post-operative	0.993	0.990 to 0.995	< 0.001**
	Last follow-up	0.994	0.991 to 0.995	< 0.001**
SO (°)	Pre-operative	0.995	0.993 to 0.997	< 0.001**
	Post-operative	0.993	0.990 to 0.995	< 0.001**
	Last follow-up	0.996	0.994 to 0.997	< 0.001**
IO (°)	Pre-operative	0.989	0.985 to 0.992	< 0.001**
	Post-operative	0.991	0.987 to 0.993	< 0.001**
	Last follow-up	0.990	0.986 to 0.993	< 0.001**
HO (°)	Pre-operative	0.978	0.969 to 0.984	< 0.001**
	Post-operative	0.989	0.985 to 0.992	< 0.001**
	Last follow-up	0.984	0.977 to 0.988	< 0.001**
Cobb angle (°)	Pre-operative	0.995	0.992 to 0.996	< 0.001**
	Post-operative	0.994	0.991 to 0.995	< 0.001**
	Last follow-up	0.996	0.994 to 0.997	< 0.001**
AVT (mm)	Pre-operative	0.994	0.991 to 0.996	< 0.001**
	Post-operative	0.995	0.993 to 0.996	< 0.001**
	Last follow-up	0.992	0.985 to 0.996	< 0.001**
CD (mm)	Pre-operative	0.988	0.984 to 0.993	< 0.001**
	Post-operative	0.992	0.989 to 0.994	< 0.001**
	Last follow-up	0.991	0.985 to 0.995	< 0.001**

HLLL: healthy lower limb length; ALLL: affected lower limb length; SO: sacral obliquity; IO: iliac obliquity; HO: hip obliquity; AVT: apical vertebral translation; CD: coronal decompensation.

According to Table 3, in the LDG, ALLL significantly increased at both postoperative and last follow-up assessments compared to preoperative measurements (pre-op vs. post-op: *P* = 0.003, pre-op vs. f-u: *P* = 0.001), with no statistical difference observed between the postoperative and last follow-up evaluations (*P*>0.05). No significant differences were found in HLLL before and after surgery and at the last follow-up (*P*>0.05). Additionally, LLLD, SO, IO, and HO showed significantly decreases at both postoperative and last follow-up assessments compared to the preoperative measurements (LLLD, pre-op vs. post-op: *P*<0.001, pre-op vs. f-u: *P*<0.001; SO, pre-op vs. post-op: *P* = 0.007, pre-op vs. f-u: *P* = 0.003; IO, pre-op vs. post-op: *P* = 0.045, pre-op vs. f-u: *P* = 0.035; HO, pre-op vs. post-op: *P* = 0.004, pre-op vs. f-u: *P* = 0.003), with no statistical difference observed between the postoperative and last follow-up evaluations (*P*>0.05). No significant differences were found in the Cobb angle, AVT, and CD before and after surgery and at the last follow-up (*P*>0.05).

**Table 3:** Comparison of pre-operative, post-operative, and last follow-up radiographic parameters in patients of the low dislocation group

Variable	Pre-operative	Post-operative	Last follow-up		*P*-value	
				Pre-op vs. Post-op	Post-op vs. F-u	Pre-op vs. F-u
HLLL (mm)	925.3 ± 51.3	925.4 ± 51.2	925.5 ± 51.3	0.773	0.739	0.635
ALLL (mm)	920.5 ± 52.1	922.5 ± 51.6	922.6 ± 51.8	0.003*	0.379	0.001*
LLLD (mm)	4.7 ± 5.7	3.0 ± 3.5	2.8 ± 3.5	< 0.001**	0.204	< 0.001**
SO (°)	3.7 ± 2.8	3.4 ± 2.3	3.4 ± 2.3	0.007*	0.074	0.003*
IO (°)	3.5 ± 3.0	3.3 ± 2.8	3.3 ± 2.8	0.045*	0.449	0.035*
HO (°)	2.6 ± 2.4	2.2 ± 2.3	2.2 ± 2.3	0.004*	0.274	0.003*
Cobb angle (°)	7.8 ± 3.1	7.8 ± 3.1	7.8 ± 3.0	0.261	0.543	0.351
AVT (mm)	9.2 ± 2.8	9.1 ± 2.8	9.2 ± 2.8	0.524	0.621	0.591
CD (mm)	11.2 ± 3.1	11.2 ± 3.3	11.2 ± 3.2	0.796	0.639	0.933

HLLL: healthy lower limb length; ALLL: affected lower limb length; LLLD: lower limb length difference; SO: sacral obliquity; IO: iliac obliquity; HO: hip obliquity; AVT: apical vertebral translation; CD: coronal decompensation. *: Significant at *P*<0.05, **: Significant at *P*<0.001 (Paired sample *t*-test).

According to Table 4, in the HDG, ALLL significantly increased at postoperative assessments compared to preoperative measurements (*P*<0.001) and at last follow-up assessments compared to both preoperative and postoperative measurements (pre-op vs. f-u: *P*<0.001, post-op vs. f-u: *P*<0.001). No significant differences were found in HLLL before and after surgery and at the last follow-up (*P*>0.05). Additionally, LLLD significantly decreased at postoperative assessments compared to preoperative measurements (*P*<0.001) and at last follow-up assessments compared to both preoperative and postoperative measurements (pre-op vs. f-u: *P*<0.001, post-op vs. f-u: *P*<0.001). Furthermore, SO, IO, and HO showed significantly decreases at both postoperative and last follow-up assessments compared to the preoperative measurements (SO, pre-op vs. post-op: *P*<0.001, pre-op vs. f-u: *P*<0.001; IO, pre-op vs. post-op: *P*<0.001, pre-op vs. f-u: *P*<0.001; HO, pre-op vs. post-op: *P*<0.001, pre-op vs. f-u: *P*<0.001), with no statistical difference observed between the postoperative and last follow-up evaluations (*P*>0.05). No significant differences were found in the Cobb angle and AVT before and after surgery and at the last follow-up (*P*>0.05), while the CD significantly decreased at postoperative assessments compared to preoperative measurements (*P* = 0.033) and at last follow-up assessments compared to both preoperative and postoperative measurements (pre-op vs. f-u: *P* = 0.017, post-op vs. f-u: *P* = 0.014).

**Table 4:** Comparison of pre-operative, post-operative, and last follow-up radiographic parameters in patients of the high dislocation group

Variable	Pre-operative	Post-operative	Last follow-up	*P*-value		
				Pre-op vs. Post-op	Post-op vs. F-u	Pre-op vs. F-u
HLLL (mm)	927.7 ± 82.3	926.5 ± 81.9	926.2 ± 82.2	0.362	0.289	0.258
ALLL (mm)	859.1 ± 77.4	889.4 ± 80.0	898.8 ± 80.4	< 0.001**	< 0.001**	< 0.001**
LLLD (mm)	68.6 ± 21.0	37.1 ± 20.0	27.4 ± 18.9	< 0.001**	< 0.001**	< 0.001**
SO (°)	14.6 ± 2.1	12.8 ± 2.8	12.6 ± 2.9	< 0.001**	0.138	< 0.001**
IO (°)	12.9 ± 2.5	11.0 ± 2.9	10.9 ± 2.8	< 0.001**	0.232	< 0.001**
HO (°)	7.0 ± 3.7	-3.3 ± 4.7	-3.3 ± 4.7	< 0.001**	0.320	< 0.001**
Cobb angle (°)	21.6 ±10.0	20.6 ± 7.5	20.3 ± 7.3	0.161	0.123	0.097
AVT (mm)	22.3 ± 9.9	21.6 ± 8.3	21.4 ± 8.0	0.255	0.150	0.135
CD (mm)	21.9 ± 6.5	19.7 ± 7.1	19.3 ± 7.3	0.033*	0.014*	0.017*

HLLL: healthy lower limb length; ALLL: affected lower limb length; LLLD: lower limb length difference; SO: sacral obliquity; IO: iliac obliquity; HO: hip obliquity; AVT: apical vertebral translation; CD: coronal decompensation. *: Significant at *P*<0.05, **: Significant at *P*<0.001 (Paired sample *t*-test).

**Fig. 2:**
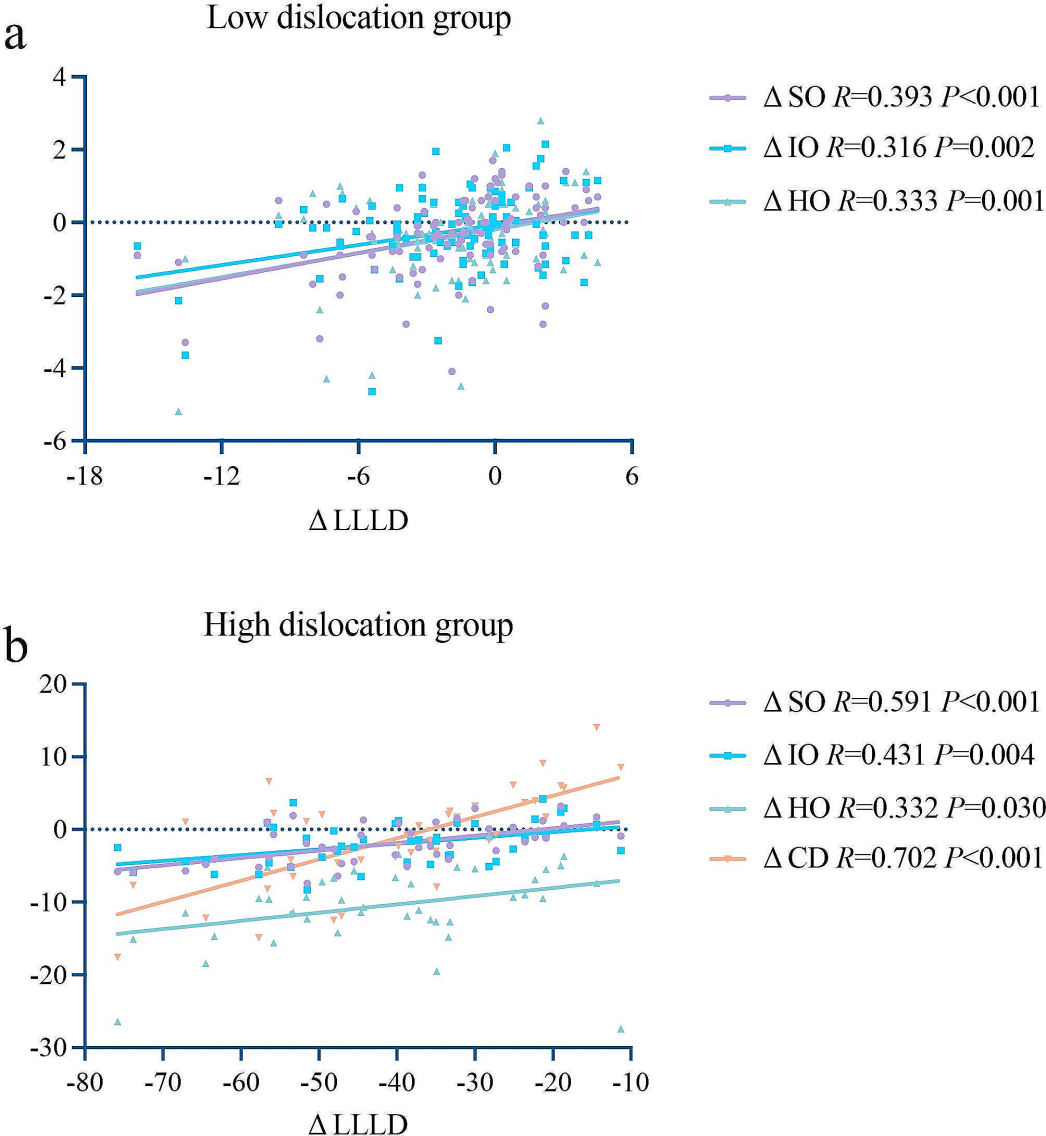
Scatter plots illustrating the correlation between the variations of lower limb length discrepancy and spinopelvic parameters in the low dislocation group and high dislocation group. **(a)** Low dislocation group; **(b)** High dislocation group

The outcomes from Table 5 revealed that there were significant differences in the variations in LLLD, SO, IO, and HO between the HDG and LDG. Specially, the variations in LLLD, SO, IO, and HO were significantly greater in the LDG compared to the HDG (LLLD: *P*<0.001; SO: *P* = 0.001, IO, *P*<0.001; HO: *P*<0.001). Moving on to Fig. 2, the variation in LLLD exhibited a significant correlation with variations in SO (*R* = 0.393, *P* < 0.001), IO (*R* = 0.316, *P* = 0.002), and HO (*R* = 0.333, *P* = 0.001) in the LDG. In the HDG, the variation in LLLD displayed a significant correlation with variations in SO (*R* = 0.591, *P* < 0.001), IO (*R* = 0.431, *P* = 0.004), HO (*R* = 0.332, *P* = 0.030), and CD (*R* = 0.702, *P* < 0.001).

**Table 5:** Comparison of variations in radiographic parameters between patients in the low dislocation group and high dislocation group

Variable	LDG (*n* = 94)	HDG (*n* = 43)	*P*-value	95% CI for difference in group means
Δ LLLD (mm)	-1.9 ± 3.8	-41.1 ± 16.1	< 0.001**	34.24 to 44.24
Δ SO (°)	-0.4 ± 1.1	-2.0 ± 2.8	0.001*	0.75 to 2.51
Δ IO (°)	-0.2 ± 1.1	-2.0 ± 3.0	< 0.001**	0.85 to 2.72
Δ HO (°)	-0.4 ± 1.2	-10.4 ± 5.5	< 0.001**	8.32 to 11.74
Δ Cobb angle (°)	0 ± 0.5	-1.3 ±4.9	0.086	-0.19 to 2.83
Δ AVT (mm)	0 ± 0.8	-0.9 ± 3.9	0.157	-0.35 to 2.06
Δ CD (mm)	0 ± 0.5	-1.5 ±6.7	0.139	-0.52 to 3.60

LDG: low dislocation group; HDG: high dislocation group; LLLD: lower limb length difference; SO: sacral obliquity; IO: iliac obliquity; HO: hip obliquity; AVT: apical vertebral translation; CD: coronal decompensation. *: Significant at *P*<0.05, **: Significant at *P*<0.001 (Independent sample *t*-test)

In terms of postoperative complications, there were 2 cases of hip dislocation in the LDG and 1 case in the HDG. These cases were successfully managed with closed reduction and cast immobilization for three weeks. Notably, there were no reported cases of infection, symptomatic deep vein thrombosis, or nerve palsy in either group.

## Discussion

This study examined the changes in LLLD, pelvic obliquity, and spinal scoliosis following THA in DDH patients, with a specific focus on understanding the impact of LLLD on spinopelvic compensation. Significant reductions in LLLD, SO, IO, and HO were observed postoperatively in both LDG and HDG. Furthermore, correlations between variation in LLLD and variations in SO, IO, and HO were noted, indicating a potential restoration of pelvic obliquity due to THA-induced LLLD reduction. Moreover, a notable decrease in postoperative CD was observed in the HDG, with its variation significantly correlated with variation in LLLD, suggesting a potential correction of spinal coronal imbalance following THA in DDH patients with high dislocation.

Initially compensating for LLLD, the pelvis demonstrates obliquity, and as the LLLD increases, the degree of pelvic rotation also significantly rises [16]. Conventionally, the pelvic structure consists of the sacrum, iliac crest, and ischium, with only two non-osseous joints: the sacroiliac joint and the symphysis pubis, both of which allow slight movement. Consequently, after the developmental phase, the entire pelvis is often perceived as a rigid, immovable bony structure. However, an increasing number of studies have proposed the existence of a compensatory mechanism within the pelvis, introducing the concepts of SO and IO to assess its ability to compensate for LLLD [11, 12]. Therefore, we evaluated the recovery of postoperative internal pelvic compensation in patients with DDH by measuring SO, IO, and HO before and after surgery. Moreover, to achieve coronal balance and maintain an upright position, the spine tends to flex towards the longer side of the legs, leading to the manifestation of scoliosis on the shorter side. A study has demonstrated that LLLD greater than 9 mm could contribute to the development of lumbar scoliosis [17]. Therefore, Cobb angle, AVT, and CD were measured before and after surgery to evaluate the effect of the change in LLLD on the degree of coronal balance restoration of the spine.

The early study highlighted a conspicuous recovery in the ALLL and a decrease in the pelvic obliquity following THA in patients with DDH [11]. In our current investigation, we observed a notable improvement in ALLL and a substantial reduction in LLLD in both the LDG and HDG following THA, signifying significant enhancements compared to preoperative measurements. Moreover, postoperative SO, IO and HO were significantly reduced in both groups compared to preoperative values. These findings robustly supported the efficacy of THA in restoring LLL and addressing pelvic compensation in DDH patients. Additionally, a previous investigation noted a gradual reduction in pelvic obliquity and LLLD over time in individuals with severe hip dislocation following THA [12]. Our present study revealed a noteworthy decrease in LLLD among patients in the HDG at the last follow-up in comparison to the immediate postoperative period. While SO, IO, and HO exhibited a decrease at the last follow-up compared to the immediate postoperative period, statistical significance was not observed. These findings suggested a positive trend toward improved lower limb symmetry and coronal pelvic alignment in the HDG over the follow-up period. This observed favorable trajectory could be attributed to the initial challenge of soft tissue adaptation to the sudden and significant alteration in LLL among patients with high dislocation immediately after surgery. As time progresses, there was a gradual adaptation of soft tissue tightness to the new bone structure, accompanied by corresponding adjustments in pelvic obliquity to some extent. Moreover, while the Cobb Angle and AVT in the HDG exhibited a decrease after surgery compared to preoperative values, no statistical significance was observed. Notably, our study identified a statistically significant reduction in CD among patients in the HDG postoperatively compared to the preoperative state, with a further significant decrease noted at the last follow-up compared to the immediate postoperative period. This suggested a substantial improvement in spinal coronal balance in patients with high dislocation after THA, with continued gradual recovery observed in the postoperative period within two years.

An inequality in LLL has been associated with the development of scoliosis, and the extent of this length difference correlates with the degree of scoliotic curvature [18]. Despite this understanding, there is a lack of research on the recovery of scoliosis in DDH patients after THA. In our study, we observed that at the last follow-up, the HDG displayed a significantly more substantial recovery in LLLD, SO, IO, and HO compared to the LDG. Additionally, the HDG also exhibited a greater degree of recovery in Cobb Angle, AVT, and CD, although statistical significance was not achieved. Therefore, our assertion was that individuals with DDH achieved considerable recovery in pelvic compensation following THA surgery, with those having high dislocation experiencing a more pronounced recovery compared to their low dislocation counterparts. Furthermore, our study primarily examined the relationship between the restoration of pelvic obliquity and scoliosis and the change in LLLD in patients with DDH after THA. Our findings revealed a mild linear correlation between the change in LLLD and the alterations in SO, IO, and HO in patients with low dislocation. In patients with high dislocation, the change in LLLD demonstrated mild linear correlation with IO and HO alterations, while exhibited a significant linear correlation with SO and CD alterations. Earlier research has established an association between severe coronal imbalance and lower back pain symptoms, as well as diminished functional scores, in individuals with scoliosis [19]. Consequently, in the context of primary THA for patients with high dislocation and notable preoperative scoliosis, our study implied that mitigating LLLD may play a role in restoring spinal coronal balance to a certain extent.

Several limitations merit consideration in our study. Firstly, the retrospective nature of our study imposes inherent constraints in data collection. Secondly, the study population was derived from a single center, which may potentially impact the generalizability of our results. Thus, it is imperative to conduct additional studies with larger, multicenter cohorts to validate and extend our results. Thirdly, it is crucial to acknowledge that THA may alter the sagittal alignment of the pelvis and spine [20]. While most coronal plane parameters do not exhibit significant correlations with sagittal plane parameters in adult scoliosis [21], the relatively modest alterations in spinopelvic parameters studied here could introduce confounding factors, making even subtle influences from the sagittal plane significant in this study.

## Conclusions

The present study investigated the impact of THA on LLLD, pelvic obliquity, and scoliosis in patients with DDH. Our findings revealed significant improvements in ALLL and a reduction in LLLD after THA, highlighting the effectiveness of THA in restoring lower limb symmetry, especially for patients with high dislocation. Additionally, postoperative measurements of SO, IO, HO, and CD showed significant reductions, indicating a restoration of spinopelvic compensation following THA. Positive correlations were identified between the variations in LLLD and spinopelvic measurements, suggesting that intraoperative adjustment of LLLD is closely associated with the recovery from pelvic obliquity and coronal imbalance of the spine after surgery.

## Data Availability

The dataset analyzed during the current study are available from the corresponding author on reasonable request.

## Abbreviations

LLLD: Lower limb length discrepancy
DDH: Developmental dysplasia of the hip
THA: Total hip arthroplasty
LDG: Low dislocation group
HDG: High dislocation group
LLL: Lower limb length
SO: Sacral obliquity
IO: Iliac obliquity
HO: Hip obliquity
AVT: Apical vertebral translation
CD: Coronal decompensation
ALLL: Affected lower limb length
HLLL: Healthy lower limb length
